# Effect of Amino Silicone Oil-Phosphorylation Hybrid Modification on the Properties of Microcellulose Fibers

**DOI:** 10.3390/polym16081123

**Published:** 2024-04-17

**Authors:** Quan Yuan, Guimei Zhang, Chunxuan Li, Shiwei Xu, Liping He

**Affiliations:** 1 State Key Laboratory of Advanced Design and Manufacturing Technology for Vehicle, Hunan University, Changsha 410082, China; quany@hnu.edu.cn; 2 College of Mechanical and Vehicle Engineering, Hunan University, Changsha 410082, China; 3 Suzhou Research Institute of Hunan University, Suzhou 215131, China; 4 Hunan Jinjian New Material Technology Co., Ltd., Yongzhou 426181, China; hunanjingjian@126.com (G.Z.); lichunxuan0508@163.com (C.L.)

**Keywords:** microcellulose fibers, amino silicone oil-phosphorylated, high hydrophobic, thermal stability, flame retardancy, self-extinguishing

## Abstract

Microcellulose materials are increasingly considered multifunctional candidates for emerging energy applications. Microcellulose fibers (MCF) are a kind of bio-based reinforcement in composites, and their hydrophilic character hinders their wide application in industry. Thus, in the present work, MCF was hybrid-modified by amino silicone oil-phosphorylated to fabricate hydrophobic, thermal stability, and flame-retardant microcellulose fibers for potential application in vehicle engineering. The results showed that the amino silicone oil-phosphorylated (ASOP) hybrid modification could transform the surface property of microcellulose from hydrophilic to hydrophobic and improve the compatibility between MCF and resin matrix. Meanwhile, the ASOP treatment led to the formation of an amino silicone oil film layer on the surface of the microcellulose, which improved the thermal stability of the MCF. Furthermore, the ASOP hybrid modification microcellulose fibers paper (100% microcellulose fibers paper) was transformed from flammable to flame-retardant and showed self-extinguishing behavior after burning under flame for 2 s. The flame-retardant mechanism was attributed to the formation of the char layer in the condensed phase and the production of non-combustible gases in the gaseous phase.

## 1. Introduction

The vehicle interiors are an important part of vehicles. Most vehicle interior parts are mainly composed of various types of polymers and their composites [1]. With the gradual improvement of safe, environmentally friendly, and comfortable vehicles, the bio-based renewable microcellulose fibers (MCF) reinforced polymer green composites have become a key development direction in the transportation industry [2] because interiors made of green composites can not only reduce the cost but also reduce Volatile organic compounds (VOCs) and improve air quality in vehicles [3,4]. Nevertheless, bio-based MCFs are highly hydrophilic due to the large number of polar hydroxyl and phenolic hydroxyl functional groups [5,6], which makes them hardly compatible with hydrophobic polymer matrices. This will further decrease the mechanical properties of microcellulose fibers-reinforced composites because of poor bonding at the interfacial [7]. Moreover, MCF is easily carbonized as a filler at high temperatures [8], which not only triggers thermal decomposition but also reduces its mechanical properties [9]. On the other hand, the flammability problem must be resolved if MCF composites are to be widely used in the field of battery pack materials for vehicles [10].

Therefore, in view of the above problems, researchers focused on cellulose modification methods to investigate the effect of ASO and phosphorylation modification on the thermal properties of cellulose so as to apply it to the interiors of vehicles. Liu Y et al. [11] prepared flame-retardant cellulose fibers by blending cellulose with ASO. It was found that ASO modification could prevent the degradation of cellulose fibers, favored char formation, and improved the flame retardancy of cellulose. He LP et al. [12] used ASO for surface modification treatment of ramie fibers and found that the modified ramie fibers hardly decomposed before 230 °C, with a residual char of 11% at 600 °C, and the heat resistance of the ASO-modified ramie fibers was greatly improved. Noguchi et al. [13] used an aqueous solution of NH_4_H_2_PO_4_ and urea for phosphorylating modification of softwood pulp, introducing phosphate groups on cellulose microfibers to obtain phosphorylated cellulose with high crystallinity and thermal stability. Suflet et al. [14] successfully synthesized water-soluble phosphorylated cellulose by reaction of microcrystalline cellulose with phosphoric acid. The samples were tested and found to have good thermal stability at 200 °C, which has a broad application scenario. B Sara and I Nicolas et al. [15,16,17] developed a method for the preparation of phosphorylated microcellulose under urea-free, corrosion-free, and gentle experimental conditions. The phosphorylated microcellulose was filled into chitosan to prepare flame-retardant microcellulose chitosan films with improved thermal properties and flame retardancy. The above studies showed that phosphorylation modification could improve the thermal properties and flame retardancy of MCF. Nonetheless, the phosphorylation-modified MCF still has a strong surface polarity, which is prone to poor melt fluidity, uneven distribution, and agglomeration of MCF during the injection molding process with the hydrophobic polymer matrix, thus reducing the interfacial bonding and mechanical properties of the composites, limiting microcellulose fibers’ application in vehicle engineering.

Therefore, a hybrid modification method of MCF by amino silicone oil and NH_4_H_2_PO_4_ was proposed to fabricate highly hydrophobic and flame-retardant bio-based reinforced microcellulose fibers. The effects of amino silicone oil-phosphorylated (ASOP) hybrid modification on the chemical structure, micro-morphology, thermal properties and flame retardancy, and surface feature of MCF were investigated by FTIR, XRD, XPS, SEM-EDX, TGA, and contact angle analysis. The aim of the present work is to investigate the synergistic effect of ASOP hybrid modification and to deeply study the mechanism of the effect of amino silicone oil (ASO) and NH_4_H_2_PO_4_ on surface features and properties of the resulting material.

## 2. Experimental

### 2.1. Materials

The MCF used in this study was wood pulp fiber supplied by Northern Century Cellulosic Materials Co., Suzhou, China. All chemicals used in the experiments were of analytical grades: ammonium dihydrogen phosphate (NH_4_H_2_PO_4_, >99%) was supplied by Sinopharm, Changsha, China, urea (urea, >99%), sodium hydroxide (NaOH, 99%) was supplied by Huihong, Changsha, China, amino silicone oil was supplied by Guangzhou Zhuangjie Chemical Co., Ltd, Guangzhou, China. and was modified in the lab. All the reagents were used directly without further purification, and deionized water was used in all reactions.

### 2.2. Methods

#### 2.2.1. ASOP Modified

The MCF was pre-treated with the ASO and subsequently treated with phosphorate to form amino silicone oil-phosphorylation hybrid modification microcellulose fibers (ASOP-MCF). The processes are described below.

Firstly, the suspension was prepared by dispersing an appropriate amount of MCF into ASO solution and placed in an ultrasonic unit for 0.5 h at 50 °C (The mass ratio of MCF to ASO is 2:1). After the reaction, it was filtered and dried to constant weight in a constant temperature electric oven at 60 °C, labeled as ASO-MCF.

**Figure 1:**
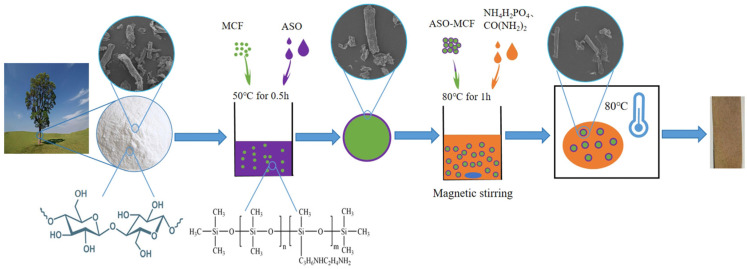
ASOP-MCF modification process.

Secondly, ASO-MCF was dispersed in distilled water to prepare a suspension of 4 wt%, and appropriate amounts of NH_4_H_2_PO_4_ and urea were added to the suspension with magnetic stirring at 80 °C for 30 min, in which ratio of anhydroglucose units of microcellulose (AGU) chain to reagents being: 1:1.2:4.9. Afterwards, the mixture was filtered and completely dried in a constant temperature electric oven dried at 105 °C. Finally, the ASOP hybrid modified materials were re-dispersed into deionized water (0.5 wt%), washed twice, and its pH was adjusted to 9.5 using 0.1 M sodium hydroxide solution, then filtered and dried to constant weight, labeled as ASOP-MCF, and the modification process is shown in Figure 1.

#### 2.2.2. Preparation of Flame-Retardant Microcellulose Paper

The ASOP-MCF was used to prepare microcellulose paper sheets as described by Zhang [18], and all microcellulose papers were dried in the drying oven at 60 °C for 1 h before testing.

### 2.3. Characterization

#### 2.3.1. Chemical Structural Analysis

(1) FTIR Spectroscopy

The FTIR spectra of microcellulose were analyzed on a Nicolet iN10 FT-IR spectrophotometer (Thermo Fisher Scientific, Waltham, MA, USA) using the KBr pellet method. All samples were recorded in 400–4000 cm^−1^ range with a resolution of 4 cm^−1^ and an accumulation of 32 scans.

(2) X-ray Diffraction (XRD) Analysis

The crystalline structure of fiber samples was characterized by XRD (D8 Advance, Bruker, Shenzhen, China) equipped with a Cu Ka radiation source (k = 0.1542 nm), and the scanning range was within the range of 2θ = 10–60°. The crystallinity index (CrI) was determined by the Segal empirical equation: CrI = (I_002_ − I_am_)/I_002_, where I_002_ is the maximum peak intensity at the (002) crystal plane, and I_am_ is the minimum peak intensity in the amorphous region at 2θ = 18.3° [19].

(3) X-ray Photoelectron Spectroscopy (XPS)

The XPS spectra were collected with a Thermo Scientific K-Alpha electron spectrometer using a monochromatic Al Kα X-ray source operated at 150 W. The substrates were positioned at an angle of 90° under an ultrahigh vacuum of less than 10^−7^ Pa. The binding energy (BE) scale was referenced to the C1s line of aliphatic carbon, set at 285.0 eV.

#### 2.3.2. Scanning Electron Microscopy-Energy Dispersive X-ray Analysis (SEM-EDX)

The microstructure of cellulose fiber was tested using a scanning electron microscope (Zeiss Sigma 300, Oberkochen, Germany). Detector Si (Li) EDAX-10 mm^2^ was used to evaluate the position of the elements phosphate and silicon in the ASOP-MCF.

#### 2.3.3. Thermo-Gravimetric and Flammability Analysis

(1) Thermo-Gravimetric Analysis (TGA)

The thermal properties of fiber samples were evaluated using a TGA (Netzsch STA 449 F3, Selber, Bavaria, Germany) in nitrogen and in air, respectively, with the samples being heated from 30 to 700 °C at a rate of 10 °C/min. An alumina crucible was used to hold 5–10 mg of microcellulose fiber powdered sample. Tonset_10%_ (the temperature at 10% of weight loss), Tonset_50%_ (the temperature at 50% of weight loss), T_max_ (the temperature at maximum rate of weight loss), and the residue at 600 °C were obtained from the measurements.

(2) Flammability Analysis

The samples were tested for vertical flammability by applying a butane flame for 2 s on the short side of the samples (20 × 100 mm). The final residual amount and burning rate of the samples were recorded. The experiments were repeated three times for each sample. The burning rate of microcellulose paper can be defined by the following equation: Burning rate = microcellulose paper burning length/burning time [20].

(3) Dynamic Contact Angle

The change of MCF contact angle with time was recorded using a DSA 100 contact angle tester (KRŰSS, Hamburg, Germany). First, the samples were fully dried and laid flat on a washed slide. Then, 10 μL of deionized water was dropped on the surface of the samples, and the contact angle of the samples was recorded for a certain time. To minimize the error, the samples were tested three times, and the average of the three tests was taken.

## 3. Results

### 3.1. Chemical and Crystal Structure Characterization

#### 3.1.1. FTIR Spectroscopy Analysis

**Figure 2:**
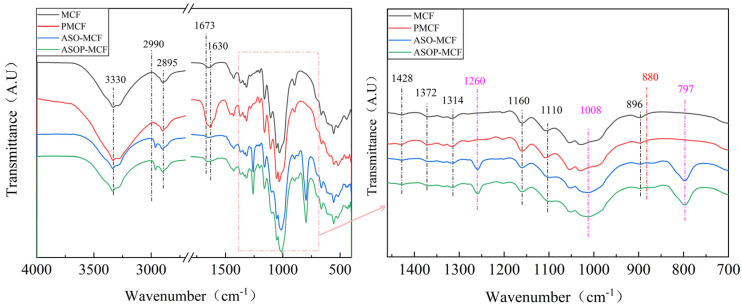
FTIR spectra of unmodified and modified microcellulose fibers.

FTIR analysis allows the assessment of the structural changes occurring at different treatment stages, and it is an effective method for demonstrating the presence of silane and phosphate groups in ASOP-MCF. As shown in Figure 2, all samples showed characteristic peaks of microcellulose fibers. Among them, the peaks in the regions of 3330 and 1630 cm^−1^ were O-H group stretching vibration and bending vibration [21,22]. The peaks at 2990, 2895, and 1673 cm^−1^ corresponded to antisymmetric stretching of the -CH_2_ group [23], C-H symmetric stretching of the aldehyde group, and C=O stretching vibration of the microcellulose chain, respectively. Notably, the C=O bonding was not observed in the MCF, which indicated that partial oxidation occurred in the modified MCF. The characteristic peaks observed at 1428, 1372, and 1314 cm^−1^ are CH_2_ symmetric bending, C-O symmetric stretching or C-H asymmetric deformation, and C-H bending of microcellulose molecules, respectively [24]. In addition, the characteristic peaks at 1160, 1110, and 896 cm^−1^ were related to C-O-C asymmetric stretching, C-O pyranose ring skeleton vibration, and C-H rocking vibration of microcellulose molecules. It is noteworthy that the characteristic peaks at 1428, 1372, 1160, and 1110 cm^−1^ were found in all the samples. At the same time, these bands belong to the characteristic of cellulose I structure [25], which is consistent with the XRD results.

Obviously, compared with MCF and ASO-MCF, the phosphorylated microcellulose showed some new peak bands at 880 cm^−1^ (seen red dotted line) that appeared as P-OH stretching vibrations [26], which indicated phosphorus-containing compounds had been grafted to the microcellulose units [27,28]. In this case, the O-H characteristic peaks of phosphorylated modified microcellulose (PMCF) at 3330 and 1630 cm^−1^ were enhanced, which was attributed to the enhanced water absorption by the introduced phosphate groups on the microcellulose surface. When modified by ASO, the FTIR of samples showed three new peak bands at 1260, 1008, and 797 cm^−1^ (See purple dotted line), which were the symmetric deformation vibration of Si-CH_3_, C-N bond, and the absorption peak of the stretching vibration of the Si-C bond in Si-CH_3_, respectively [29,30]. Additionally, the -OH characteristic peak of the modified fibers was reduced. These findings indicate that chemical reactions occurred between ASO and microcellulose during modification.

#### 3.1.2. X-ray Diffraction Analysis

**Figure 3:**
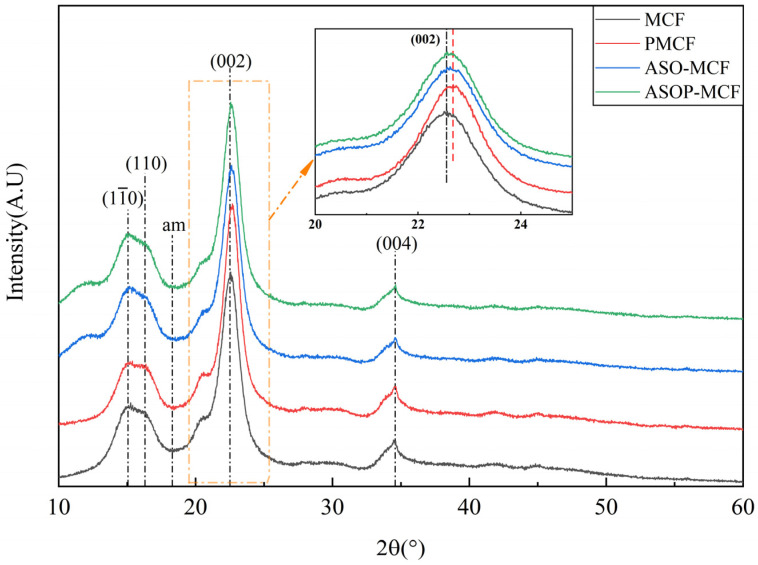
XRD pattern of microcellulose fibers before and after modification.

The crystal structure and crystallinity index of all the samples (MCF, ASO-MCF, PMCF, and ASOP-MCF) were tested and analyzed using XRD. All samples exhibited four diffraction peaks at 2θ = 15.0, 16.4, 22.6, and 34.5° corresponding to the crystallographic planes of cellulose I structure (1$\bar{1}$0), (110), (002) and (004), respectively [31,32,33], and the peaks were shifted to the right at (002) after modification of microcellulose. as shown in Figure 3. These results clearly indicated that the modifier species did not change the crystal structure of the microcellulose molecules. 

The crystallinity index of MCF was calculated to be 79.9%, which has a high degree of crystallinity and retains the more complete crystal structure of micrcellulose. The CrI of PMCF was 84.1%, which was higher than that of PMCF and MCC extracted from reed by Benhamou et al. [33] and Yu et al. [34]. In contrast to the MCF, PMCF exhibited a higher peak, and the crystallinity index increased by 6%. It was attributed to the fact that phosphorylation modification removed part of the amorphous regions of microcellulose and induced the rearrangement of the crystalline regions into a more orderly structure [35], thereby improving the crystallinity of PMCF.

It can be observed from Table 1 that the CrI of ASO-MCF is 80.1%, which is similar to the crystallinity index of MCF. This fact indicated that the effect of ASO on the crystallinity of MCF is minimal. The crystallinity index of 81.2 for ASOP-MCF decreased by about 4.29% compared to PMCF.

#### 3.1.3. XPS Analysis

**Figure 4:**
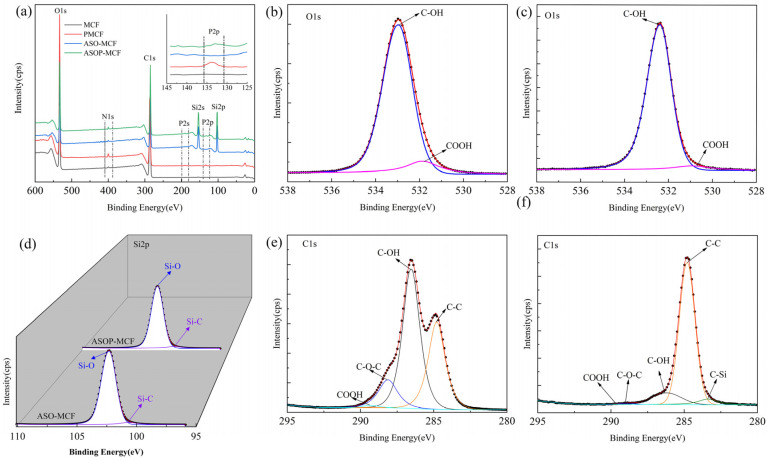
XPS spectra of microcellulose fibers: (**a**) full spectrum; MCF of (**b**) O1s, (**e**) C1s; (**d**) Si2p; ASOP-MCF of (**c**) O1s, (**f**) C1s.

The XPS spectra of microcellulose fibers before and after modification are shown in Figure 4. MCF presented two distinct peaks of elements C1s and O1s (Figure 4a) [36]. The ASOP-MCF appeared five peaks at 102.1, 133.6, 153.5, 190.5, and 400.7 eV belonging to Si2p, P2p, Si2s, P2s, and N1s, separately [36,37]. The appearance of elemental P and Si peaks strongly supported the hypothesis of the success of ASOP in modifying the microcellulose. The N1s species were probably NH^4+^ groups of NH_4_H_2_PO_4_ or air.

For ASOP-MCF, the high-resolution scan XPS spectra about O1s, Si2p, and C1s were further analyzed. As shown in Figure 4b,c, the O1s spectrum of MCF has a strong peak at 533.0 eV belonging to C-OH [38]. However, 533.0 eV can also be the peak band of the P-O-C group [38,39], and this peak seems to overlap with the peak associated with the C-OH group in the PMCF spectrum. After modification by ASO, the intensity of the C-OH group decreased due to the fact that the microcellulose surface may be covered by Si-C and Si-O groups. There was a shoulder peak at 531.3 eV assigned to the COOH group, for which the intensity was greater in the PMCF [38], as shown in Figure S1a.

The XPS spectra results of Si2p showed that the ASO-modified fibers had two main peaks. The first peak ranging from 100.4 to 100.6 eV represents a Si-C. The second peak is at 102.3–102.4 eV, which is a Si-O bond [37], as shown in Figure 4d.

The C1s spectra of MCF and ASOP-MCF are presented in Figure 4e and f, respectively. The C1s spectrum of MCF was divided into four characteristic peaks at 284.8, 286.5, 288.2, and 289.4 eV, responding for C-C, C-OH, C-O-C, and COOH groups, respectively, as shown in Figure 4e. With the abundant -OH groups on the fiber structure, the area of C-OH was larger than all the other peaks [36,37,38]. Compared to MCF, the intensity of the COOH bond of PMCF increased (Figure S1b), which was due to the partial oxidation of MCF. The samples modified by ASO appeared at a new peak at 283.3 eV, owing to the production of the C-Si bond between the MCF and ASO solution [37], as depicted in Figure 4f and Figure S1c.

### 3.2. Morphological Characterization

#### 3.2.1. Morphological Analysis

**Figure 5:**
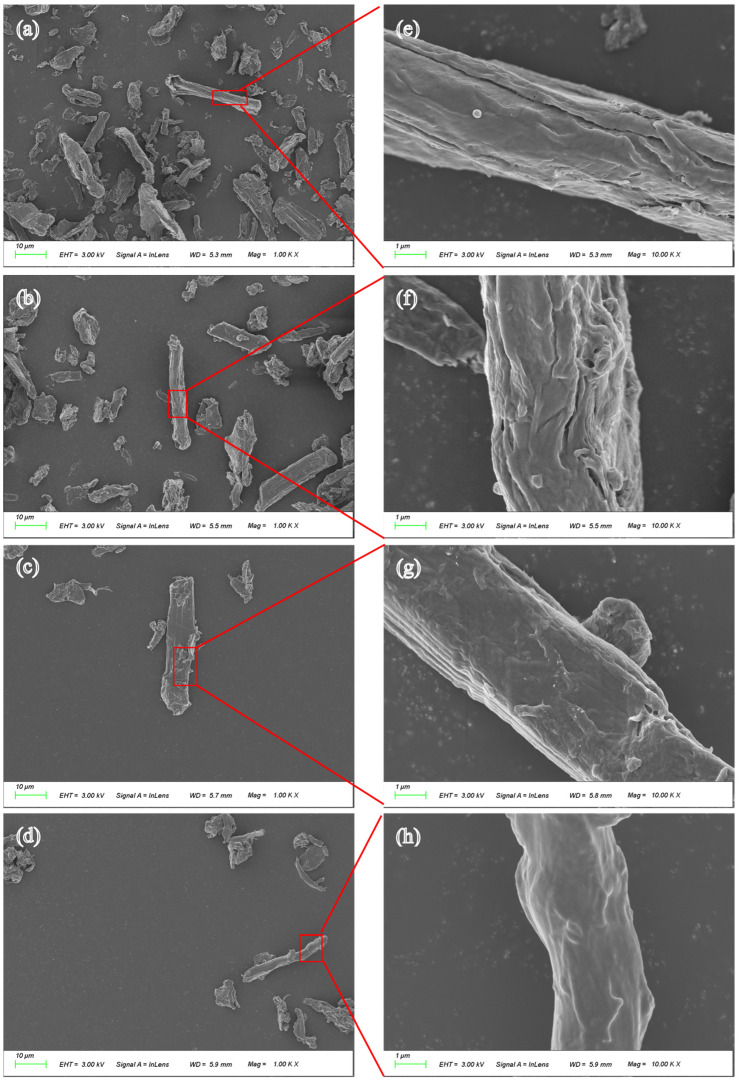
SEM image of microcellulose fibers: (**a**) MCF; (**b**) PMCF; (**c**) ASO-MCF; (**d**) ASOP-MCF; (**e**–**h**) are partially enlarged views.

**Figure 6:**
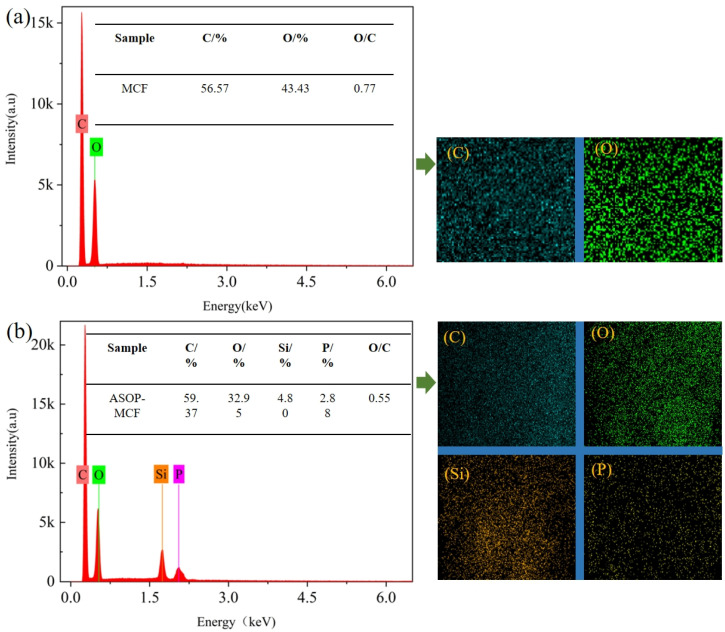
EDX of microcellulose fibers: (**a**) MCF; (**b**) ASOP-MCF.

To investigate the micromorphology and elemental composition of fibers, MCF, PMCF, ASO-MCF, and ASOP-MCF were detected by SEM and EDX (Figure 5 and Figure 6). It could be observed that MCF had a high aspect ratio, mostly in the form of elongated strips with an average diameter of 10–15 μm, and its surface was rough with obvious cracks (Figure 5a,b). After phosphorylation modification, the surface roughness of PMCF further increased with obvious cracks and pores, so PMCF had a stronger adsorption capacity for moisture and hydrophilicity. After ASO modification, the surface of the ASO-MCF cracks and grooves disappeared, and the surface became smoother (Figure 5g,h). What is more, the surface morphology of ASOP-MCF samples was smoother and denser.

#### 3.2.2. EDX Analysis

Figure 6 showed that C was the most abundant element in the tested samples, accounting for more than 50%. Within that, MCF only contained C and O elements without P and Si elements (Figure 6a). There were large amounts of P and Si elements in ASOP-MCF with percentages of 4.80% and 2.88%, respectively, and it completely covered the surface of the sample (Figure 6b). Besides, the O/C of ASOP-MCF was lower than MCF, demonstrating the lower polarity and better hydrophobicity of ASOP-MCF, which coincided with the results of the contact angle test.

### 3.3. Thermal Stability and Flame Retardancy

#### 3.3.1. Thermal Stability Analysis

**Figure 7:**
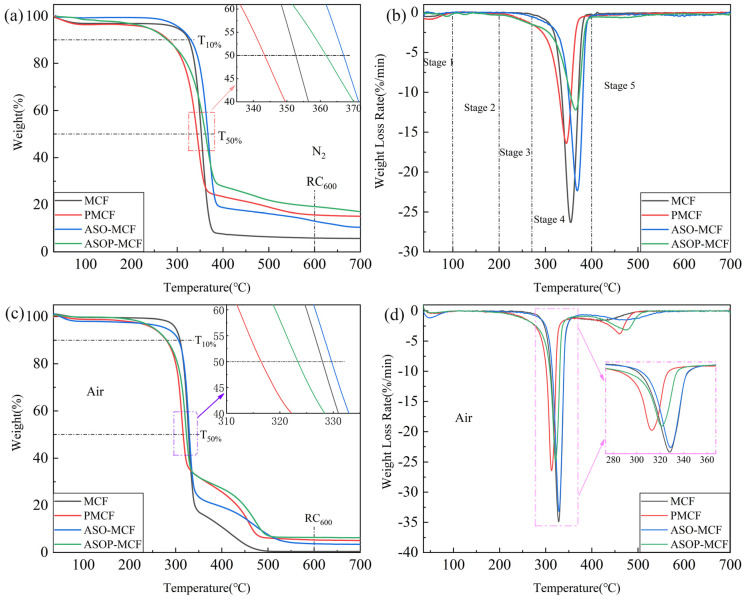
TGA (**a**,**c**) and DTG (**b**,**d**) curves of unmodified and modified microcellulose fibers under N_2_ and air atmosphere.

The whole thermal degradation and thermo-oxidative processes of MCF under N_2_ and air were described by TG and DTG (Figure 7). The decomposition of microcellulose in the N_2_ atmosphere occurs in one of the main losses that can be categorized into low and high temperatures. At a lower temperature, the glycosyl units decompose into char. Conversely, at higher temperatures, these units depolymerize into volatile products [40]. Figure 7a,b shows that the thermal stability of the samples could be divided into five stages: the first stage is 0–100 °C, caused by the evaporation of adsorbed water in the samples; The second stage is 100–200 °C, where the samples produce little decomposition and are thermal stability; The third stage is 200–270 °C, in which thermal degradation of small molecules (phosphorus-containing groups); And the fourth stage is 270–400 °C, which is the main decomposition stage, the weight loss degradation rate of samples is over 50%. There is an obvious degradation peak on the DTG curve, and the decomposition rate of microcellulose also reaches the maximum, as shown in Figure 7b. The fifth stage is 400–700 °C, and the thermal decomposition is basically finished here, with a lower weight loss rate. The detailed data referring to TG in nitrogen and air were collected in Table 2.

The T_10%_, T_50%_, and T_max_ of MCF and PMCF are 325.1 °C, 352.7 °C, 354.5 °C, and 262.4 °C, 360.4 °C, and 367.9 °C, respectively (Figure 7a). Compared to MCF, the lower T_10%_ of PMCF was attributed to the fact that the presence of phosphate groups facilitated early dehydration toward char formation and significantly the reduction in the microcellulose decomposition temperature and degradation [20,38]. The presence of phosphate groups can be explained by FTIR. Additionally, as presented in Table 2, the rate of residual char for PMCF at 600 °C was 15.6%, being 2.64 times higher than that of MCF. The increased residual char rate illustrated that the introduction of phosphorus to fibers changed the thermal degradation process.

It can be seen that the temperatures of ASO-MCF at T_10%_, T_50%,_ and T_max_ (330.0 °C, 366.8 °C and 368.7 °C) were higher than that of MCF, and the residual char rate of ASO-MCF at 600 °C was 13.1% (Table 2). It was due to the fact that the amino silicone oil forms a “protective film” as a heat-resistant protective layer encapsulated on the surface of the MCF, which substantially improved the thermal stability of the ASO-MCF.

And the temperatures of ASOP-MCF at T_50%_ and T_max_ (361.9 °C, 365.7 °C) were higher than that of PMCF (352.7 °C, 354.5 °C), and the residual char rate at 600 °C was 19.2%, with 3.3 times of that of MCF. Hence the hybrid modification of ASO and NH_4_H_2_PO_4_ could improve the thermal stability of ASOP-MCF.

As displayed in Figure 7c,d, the thermo-oxidative degradation of unmodified and modified MCF in air is divided into two steps: firstly, the formation of aliphatic char and volatiles substances, and secondly, the conversion of aliphatic char into aromatic char with the emission of CO and CO_2_ as a result of the simultaneous char formation and carbonization [41]. As in the nitrogen atmosphere, the introduction of phosphorus promoted earlier dehydration (char formation) and lower decomposition temperature compared to MCF, as shown by the T_10%_, T_50%,_ and T_max1_ values in Table 2. Besides, the char created within the first degradation step of ASOP-MCF had higher thermal stability compared to MCF, as supported by the T_max2_ values in Table 2 (T_max2_ increased from 420.0 °C to 475.3 °C), with a clearly higher amount of residual char produced at 600 °C.

#### 3.3.2. Flammability Testing

**Figure 8:**
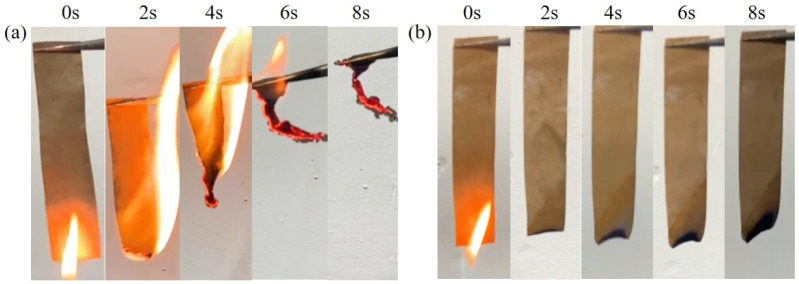
Burning of (**a**) MCF paper and (**b**) ASOP-MCF paper.

In order to investigate the combustion behavior of ASOP-MCF, vertical combustion experiments were conducted on ASOP-MCF paper samples (100% microcellulose fibers), where both control and treated 100% microcellulose fibers paper were ignited for 2 s (as shown in Figure S2). The comparison was made by comparing the flame spread time and the amount of final residue left after combustion (as shown in Table 3). Figure 8 shows photographs taken of the MCF paper before and after modification within 8 s of the flammability test, with video recordings of the tests shown in the Supporting Video Information. When a flame was applied to the MCF paper, the flame spread rapidly and burned completely within 7 s (burning rate of 1.14 cm/s, see Figure S2 and Table 3). In contrast, the paper burned slower (0.8 cm/s), and the residue increased after the flame was sprayed on the ASO-MCF paper. Obviously, when flames were applied to PMCF and ASOP-MCF papers, they burned very slowly (burning speeds of 2.67 cm/s and 3.95 cm/s, respectively), and the samples self-extinguished when the flame was removed. Even after several ignitions of the sample, PMCF and ASOP-MCF papers prevented the flame from spreading and self-extinguished. Remarkably, ASOP-MCF paper was virtually incombustible after the application of flame and completely resisting burning and flame propagation with a final residue rate of 93.7%.

### 3.4. Hydrophilic Analysis

**Figure 9:**
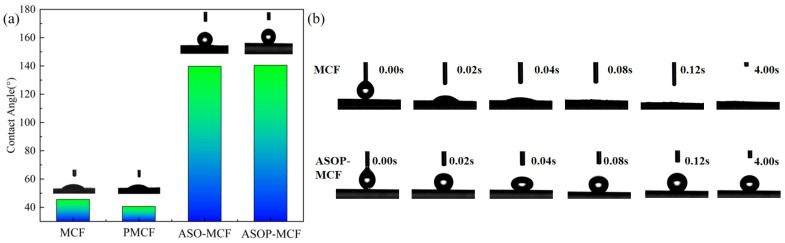
(**a**) Microcellulose fibers contact angle, (**b**) contact angle versus time.

The hydrophilicity of microcellulose is an important parameter limiting its application in the field of polymeric materials. The hydrophilicity of MCF is characterized by a dynamic contact angle test. As revealed in Figure 9a, the initial contact angle of MCF was 45.6°. It is clear that the water droplets on the fiber surface were rapidly absorbed, and the MCF shows obvious hydrophilicity. After phosphorylation modification, the contact angle of PMCF was slightly decreased to 40.6°. However, the contact angle of ASO-MCF and ASOP-MCF increased from 45.6° to 139.8° and 140.5°, respectively, after being treated with amino silicone oil (Figure 9b). Obviously, the retention time of the water droplet was longer when the deionized droplet was dropped onto the surface of the modified MCF. This indicates that the ASO modification can reduce the surface polarity of MCF and improve the hydrophobicity.

## 4. Discussion

### 4.1. Chemical and Crystal Structure of Microcellulose

The appearance of P-OH bonds in the FT-IR spectra of ASOP-MCF confirmed the success of phosphorylation. However, P- H bonds are not observed in the FT-IR spectra, which demonstrates that the majority of phosphorus on the microcellulose exists in the form of two acidic protons [38]. Additionally, ASO would form an oil film encapsulating the -OH on the surface of microcellulose, which had a certain shielding effect and weakened the intensity of the hydroxyl group [29]. 

XRD studies showed that the ASOP hybrid modification decreased the crystallinity of microcellulose. On the one hand, the ASOP hybrid modification not only occurred in the amorphous region but also at the edge of the crystalline region. On the other hand, the partial decomposition of the hydrogen bonding system in microcellulose led to an increase in the number of hydroxyl groups in the weaker hydrogen bonds [42], thus extending the amorphous region. Although the ASOP hybrid modification had a certain impact on the crystallinity of MCF, it did not change the cellulose I crystalline structure. The retention of the crystalline structure of modified MCF is of great significance in achieving the reinforcement of polypropylene composites.

From the FTIR and XPS spectra, it was observed that a sharp C=O peak appeared at 1673 cm^−1^ in the FTIR spectrum, and the intensity of the COOH band increased in the XPS spectrum of PMCF (Figure S1b) of the PMCF, which indicated that the fibers were partially oxidized during the phosphorylation. In this case, O=C-O functionalities were generated on the modified fibers in addition to the attachment of phosphate. The decreased intensity of the C-OH band of ASOP-MCF was attributed to the shielding of the partially -OH on the surface of microcellulose by the amino silicone oil film, compared to MCF and PMCF.

### 4.2. Morphological of Microcellulose

The smoother and denser surface morphology of ASOP-MCF samples, compared to MCF. It could be explained by the formation of a layer of amino-silicone oil film on the surface of microcellulose after modification with ASO, which was able to fill the cracks and grooves on the fiber’s surface. Combined with the results of the contact angle test, the amino silicone oil film could transform the surface of microcellulose fibers from hydrophilic to hydrophobic, thus improving the interfacial compatibility between MCF and polymer. Simultaneously, EDX studies showed that P and Si elements were uniformly dispersed on the fiber’s surface, which further confirmed the success of ASOP hybrid modification. 

### 4.3. Thermal and Flame-Retardant Performances

Compared with MCF, ASOP-MCF exhibited higher thermal and flame-retardant properties. Firstly, the phosphate groups adsorbed on the fiber’s surface could catalyze the dehydration of microcellulose to form char at high temperatures and prevent the thermal decomposition of microcellulose. Secondly, the ASO modification would cover an amino silicone oil film on the surface, which resulted in the formation of a double covering layer of “ASO protective film + phosphate group” on its surface and thus improved the heat resistance of microcellulose itself. When the temperature was increased, the microcellulose underwent pyrolysis, and the heterogeneous bonds on the molecular chain were broken, thereby generating the highly volatile -levoglucan, which was the 1,6-anhydro ring formed from glucose [43]. Thus, the depolymerization of microcellulose is critically linked to the instability of the C6-OH group. If the phosphate groups of the ASOP hybrid modification are mainly attached to the C6-OH, the C6 site will be protected, the production of levoglucan during pyrolysis will be inhibited, then the degradation of ASOP-MCF will be retarded or blocked.

**Figure 10:**
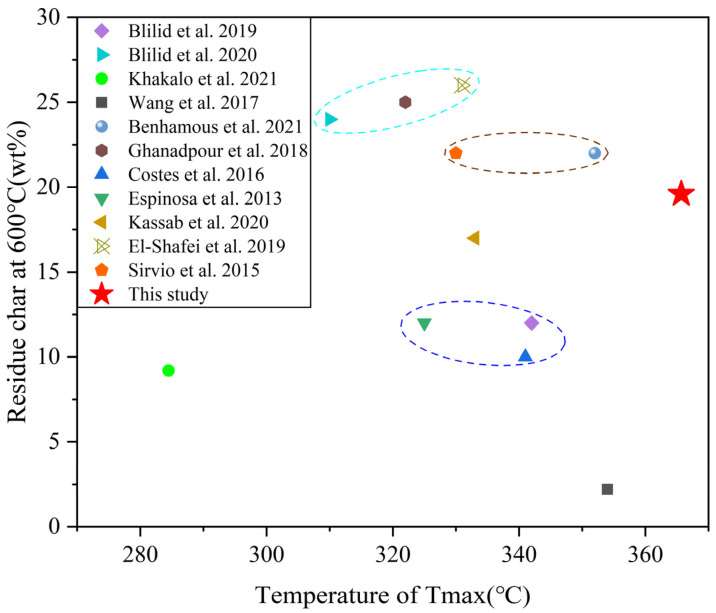
Comparison of thermal stability of ASOP-MCF in this work with other phosphorylated cellulose in the literature (Blilid et al. [15], Blilid et al. [16], Khakalo et al. [20],Wang et al. [26], Benhamous et al. [33], Ghanadpour et al. [43], Costes et al. [44], Espinosa et al. [45], Kassab et al. [46], El-Shafei et al. [47], Sirvio et al. [48]).

In order to investigate the thermal stability of ASOP-MCF, we have summarized the thermal stability of phosphorylated cellulose in this work and in the literature. As shown in Figure 10, the present work have a larger T_max_ and a comparable residual char, compared to other results in the literature, indicating that ASOP-MCF had a better thermal stability and char formation capability.

Furthermore, the lower thermo-oxidative degradation temperature of ASOP-MCF compared to MCF was also attributed to the presence of phosphorus, which catalyzes the earlier dehydration of microcellulose into char to form aromatic carbonaceous structures. All these meant that when subjected to flame, the ASOP-MCF paper could rapidly form thermally stable char under the double coverage of “ASO protective film + phosphate groups”, inhibiting the generation of volatile components and isolating the flame from contact with the interior of the material, thus producing a self-extinguishing behavior during flammability testing. 

### 4.4. Hydrophilic Performances

MCF is highly hydrophilic, and its water adsorption process is mainly achieved through the force between microcellulose molecules and water molecules. The molecular structure of MCF contains a large number of hydroxyl groups, which can easily form hydrogen bonds with water molecules and thus adsorb a significant quantity of water. Additionally, MCF with a large specific surface area can provide more adsorption space and enhance the water absorption capacity. After phosphorylation, the contact angle of PMCF was slightly reduced, which was attributed to the production of polar acidic groups P(O)(OH)2 during surface functionalization and the possible growth of the polyphosphate layer on the surface of the biocomposite [16]. The surface of microcellulose changed from hydrophilic to hydrophobic after amino silicone oil treatment, probably due to the formation of hydrogen bonds between -NH_2_ of ASO and -OH on the surface of MCF, prompting the hydrophobic alkyl groups of the amino silicone oil molecular chain tending to project outward [29,49], and improving the hydrophobicity of MCF. The contact angles of cellulose prepared by different modification methods are listed in Table 4. The ASOP hybrid modification improves the hydrophobicity of microcellulose better than other modification methods.

### 4.5. Modification Mechanism

**Figure 11:**
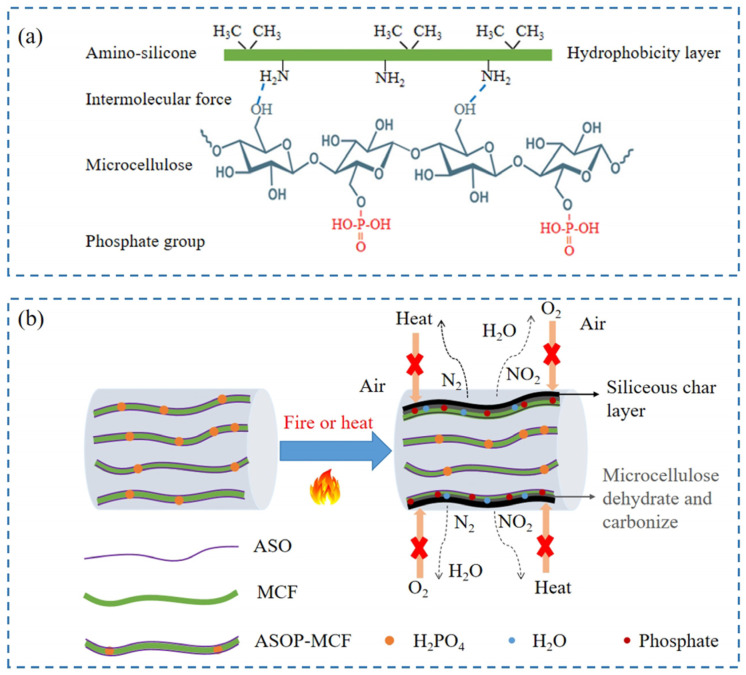
The synergistic effect of ASOP hybrid modification: (**a**) ASOP hybrid modification mechanism; (**b**) flame-retardant mechanism diagram of ASOP-MCF during burning.

The ASOP hybrid modification mechanism is shown in Figure 11a. Firstly, microcellulose adsorbs ASO on the surface through intermolecular forces. During the adsorption process, the hydroxyl group on the surface of microcellulose interacted with the amino group in the ASO molecule to form two kinds of hydrogen bonds of -O-H---N and -N-H---O, and the hydrophobic alkyl group of the amino silicone oil molecular chain tend to protrude outward [29]. Consequently, the hydrophobicity of microcellulose is enhanced. Secondly, during the process of phosphorylation modification, the phosphate group would replace the free hydroxyl group on the surface of microcellulose, thereby introducing the phosphate functional group on its surface, resulting in the enhancement of the flame-retardant ability of fibers. Therefore, the ASOP hybrid modification not only improves the interfacial compatibility between MCF and polymer but also enhances the flame retardancy of microcellulose fibers.

Based on the above discussion, we proposed the flame-retardant mechanism for ASOP-MCF, illustrated in Figure 11b. When ASOP-MCF encounters flame, ASO would be thermally degraded. The siliceous char layer produced by the decomposition of microcellulose and ASO could effectively isolate oxygen and prevent continuous combustion. Moreover, amounts of non-combustible gases such as N_2_ and NO_2_ were released from ASO combustion, which diluted the oxygen concentration [11]. Secondly, during the combustion process, ASOP-MCF decomposed and generated small molecule phosphate at high temperature, which promotes the dehydration and carbonization of the microcellulose main chain and then forms a a dense char layer to block the flame spread. Concurrently, the thermal decomposition and dehydration of the phosphate groups released a certain amount of water molecules and other gases [59], which further diluted the concentration of oxygen. Therefore, the siliceous char layer and the char formed by the dehydration of microcellulose acted as a double protective barrier, effectively inhibiting the continuous burning of the microcellulose chain via intercepting the external heat and oxygen, thereby improving the flame retardancy of MCF.

## 5. Conclusions

In this paper, the highly hydrophobic, thermal stability, and flame-retardant bio-based reinforced microcellulose fibers were prepared by hybrid modification with ASO and NH_4_H_2_PO_4_. The results of FTIR and XPS analyses showed that amino silicone oil and phosphorylated groups were successfully introduced into microcellulose. Compared with MCF, the ASOP hybrid modification could change the surface of microcellulose from hydrophilic to hydrophobic (contact angle from 45.6° to 140.5°), which improved the interfacial bonding between MCF and hydrophobic polymers and significantly improved the thermal and flame-retardant properties of MCF. Additionally, unmodified microcellulose fiber paper burned out within 7 s after applying a flame. Whereas 100% microcellulose fibers paper prepared by the ASOP hybrid modification burned slowly (with a burning rate of 3.95 cm/s), and the flame was self-extinguished after the removal of the ignition source, with a high residual amount of 93.7%. The proposed flame-retardant mechanism was ascribed to the formation of a protective layer of hybrid char layer and releasing some non-combustible volatiles. These results clearly indicated that the ASOP hybrid modified MCF was a highly hydrophobic bio-based flame-retardant material with better application potential in automotive and coating fields.

## Figures and Tables

**Table 1:** Crystallinity parameters of microcellulose fibers.

Sample	2θ(°)				CrI
	(1 $\bar{1}$0)	(110)	(002)	(004)	
MCF	14.99	16.30	22.50	34.46	79.9
PMCF	15.28	16.38	22.68	34.61	85.9
ASO-MCF	15.18	16.38	22.61	34.57	80.1
ASOP-MCF	15.15	16.32	22.58	34.59	81.2

**Table 2:** The key data was obtained from the TGA test of microcellulose fibers.

Sample	N2				Air				
	T_10%_/°C	T_50%_/°C	T_max_/°C	RC_600_/wt%	T_10%_/°C	T_50%_/°C	T_max1_/°C	T_max2_/°C	RC_600_/wt%
MCF	325.1	351.7	353.1	5.9	309.3	327.8	327.9	420.0	0.38
PMCF	300.3	346.2	345.1	13.36	281.2	316.7	312.5	458.6	5.26
ASO-MCF	330.0	366.8	368.7	13.1	306.2	329.6	328.8	481.1	3.68
ASOP-MCF	278.7	361.9	365.7	19.2	280.3	323.4	321.3	475.3	6.36

**Table 3:** The burning rate and residual amount after burning of microcellulose fibers paper.

Sample	Burning Rate, cm/s	Residue after Burning, wt%
MCF	1.14	4.2
ASO-MCF	0.8	15.3
PMCF	2.67	80.1
ASOP-MCF	3.95	93.7

**Table 4:** Contact angles of cellulose with different modification methods.

Modification Methods	Contact Angle (°)	References
Phosphorylated and chitosan modified microcellulose	93.4	Blilid [16]
Textile binder modified Microcrystalline Cellulose	99.67	Messiry [50]
UV-light curable modified cellulose and microcrystalline cellulose	111.1, 108.2	Cataldi [51,52]
Polybutylene adipate terephthalate modified microcellulose	83.1	Basil [53]
Oleic acid modified microcellulose	130.0	Huang [54]
TEMPO and MTMS modified cellulose	128.5	Sanguanwong [55]
Methyltrimethoxysilane (MTMS) modified cellulose	126.8	Gupta [56]
2, 4-toluene diisocyanate and 4,4′-diphenylmethane diisocyanate modified cellulose	131.3	Chen [57]
Quaternary ammonium salt (QAS) modified cellulose	97.3	Li [58]
ASOP modified microcellulose	140.5	This work

## Data Availability

The raw and processed data required to reproduce these findings cannot be shared at this moment due to technical and time limitations.

## Supplementary Materials

The following supporting information can be downloaded at: https://www.mdpi.com/article/10.3390/polym16081123/s1, Figure S1: XPS spectra of microcellulose fibers: (a) O1s; (b) C1s of PMCF; (c) C1s of ASO-MCF; Figure S2: Vertical flammability tests of microcellulose fibers paper before and after burning; Video S1: Vertical flammability test of paper made from MCF; Video S2: Vertical flammability test of paper made from ASO-MCF; Video S3: Vertical flammability test of paper made from PMCF; Video S4: Vertical flammability test of paper made from ASOP-MCF.
