# Supplementary material for: Effect of Amino Silicone Oil-Phosphorylation Hybrid Modification on the Properties of Microcellulose Fibers
Source: Polymers (Basel). 2024 Apr 17;16(8):1123. doi: 10.3390/polym16081123 (PMC11053708; doi:10.3390/polym16081123)
Supplement: Supplementary file 1 [file polymers-16-01123-s001.zip › polymers-2929025-supplementary.pdf]

Supplementary Information

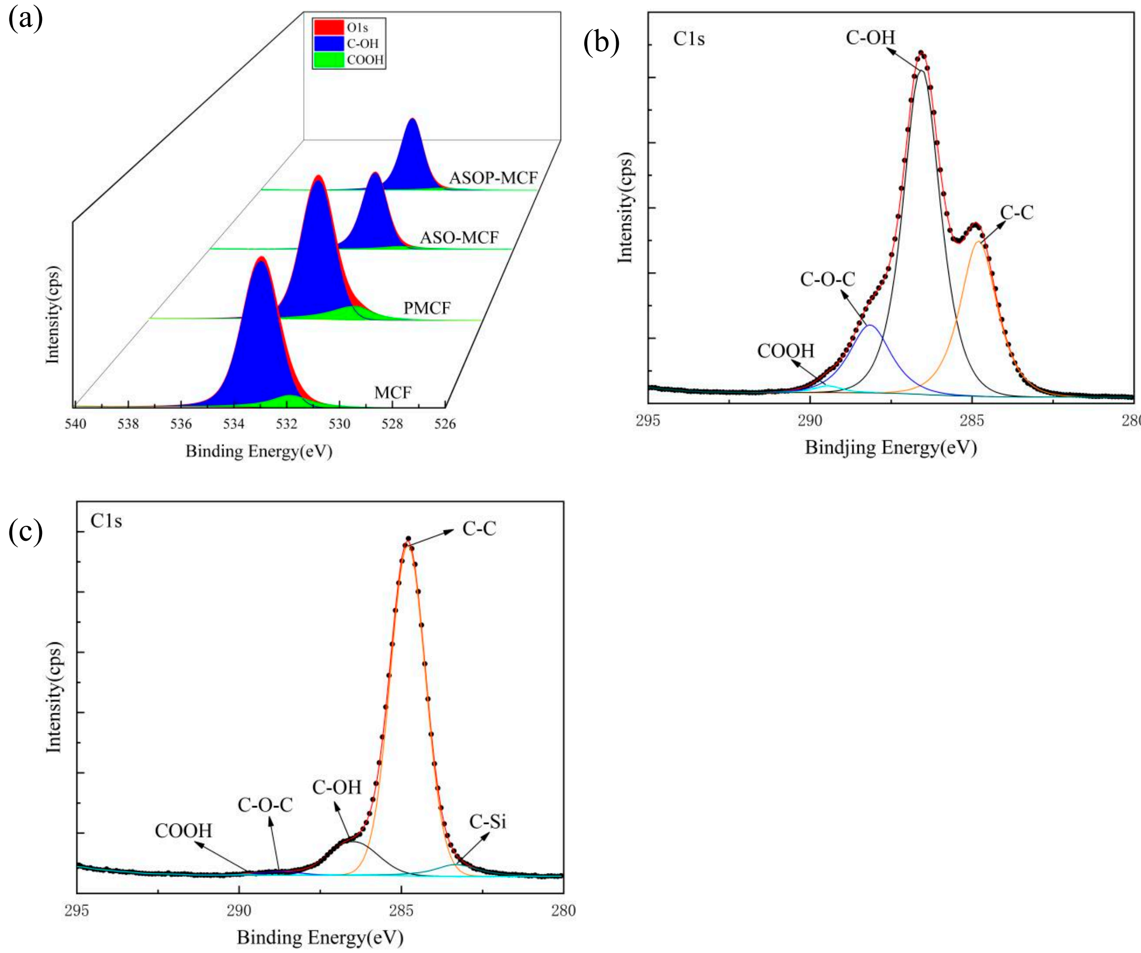

Figure S1. XPS spectra of microcellulose fibers: (a) O1s; (b) C1s of PMCF; (c) C1s of ASO-MCF.

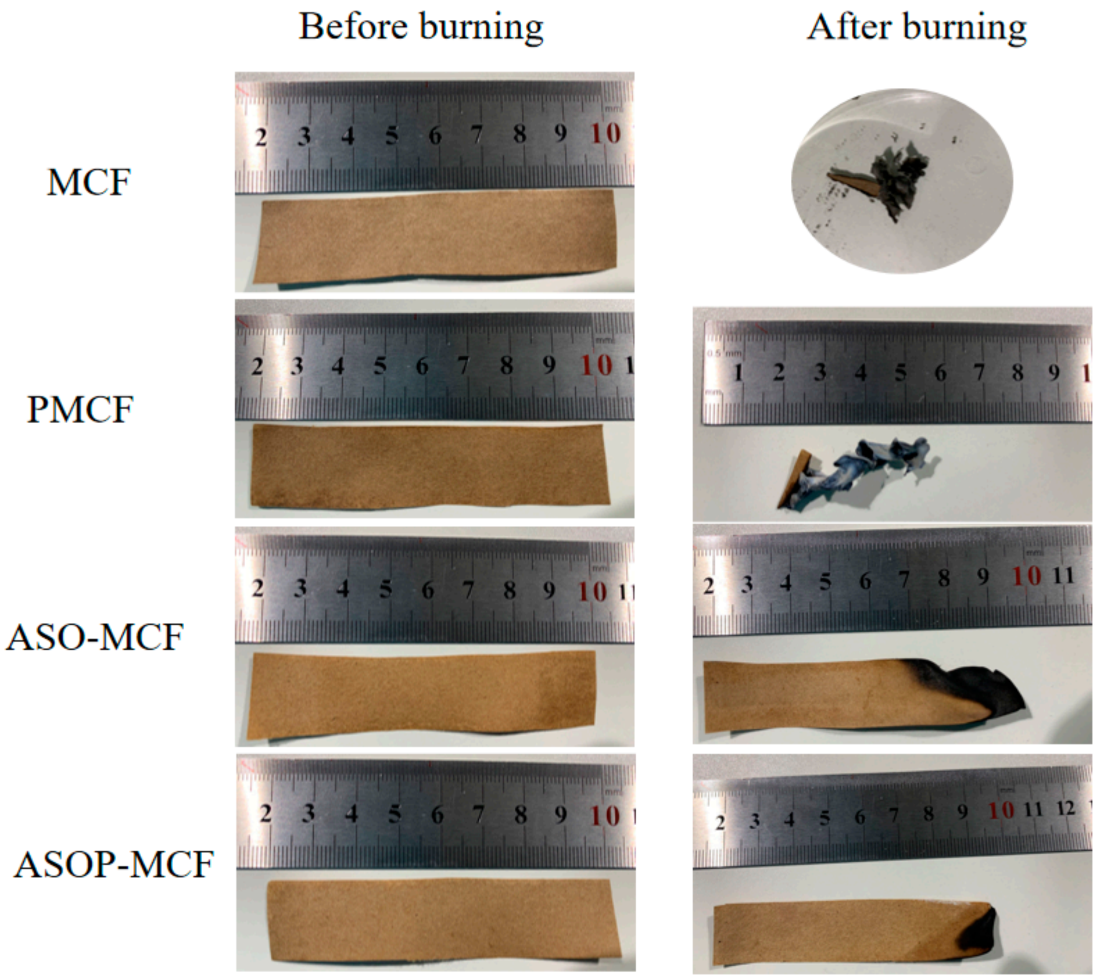

Figure S2. Vertical flammability tests of microcellulose fibers paper before and after burning.
